# Rostro-Caudal Inhibition of Hindlimb Movements in the Spinal Cord of Mice

**DOI:** 10.1371/journal.pone.0100865

**Published:** 2014-06-25

**Authors:** Vittorio Caggiano, Mirganka Sur, Emilio Bizzi

**Affiliations:** 1 McGovern Institute for Brain Research, Massachusetts Institute of Technology (MIT), Cambridge, Massachusetts, United States of America; 2 Picower Institute for Learning and Memory, Massachusetts Institute of Technology (MIT), Cambridge, Massachusetts, United States of America; Hertie Institute for Clinical Brain Research, University of Tuebingen, Germany

## Abstract

Inhibitory neurons in the adult mammalian spinal cord are known to locally modulate afferent feedback - from muscle proprioceptors and from skin receptors - to pattern motor activity for locomotion and postural control. Here, using optogenetic tools, we explored how the same population of inhibitory interneurons globally affects hindlimb movements in the spinal cord of both anesthetized and freely moving mice. Activation of inhibitory interneurons up to the middle/lower spinal cord i.e. T8–T9, were able to completely and globally suppress all ipsilateral hindlimb movements. Furthermore, the same population of interneurons - which inhibited movements - did not significantly change the sensory and proprioceptive information from the affected limbs to the cortex. These results suggest a rostro-caudal organization of inhibition in the spinal cord motor output without modulation of ascending sensory pathways.

## Introduction

Glycine and γ-aminobutyric acid (GABA) are the main inhibitory neurotransmitters in the adult mammalian spinal cord. Several lines of research have supported the idea that inhibitory interneurons have a local organization [1] defined by sensory terminals [2] to control motor neurons for limb alternation/coordination [3] and for regulation of sensory-motor reflexes [4]. At the same time, inhibitory interneurons receive inputs from primary afferents and they modulate the inflow of peripheral sensory information to spinal and supraspinal regions [5].

The local organization of inhibitory interneurons [1], [4] would suggest that these interneurons are spatially organized into functionally modular circuits similar to those observed for motorneurons [6] or for interneurons activated by NMDA agonist [7], [8]. Nevertheless, anatomical connections [9], [10], [11] and evidence of inhibition in the higher lumbar segment in ex-vivo spinal cords [12] and for breathing functions in decerebrated animals [13] suggest also the presence of a more wide spread action of spinal inhibitory neurons.

In order to understand the role and the organization of inhibitory interneurons in the control of motor and sensory information within the spinal cord with both higher precision in space and in time, we optogenetically manipulate the spinal circuits in transgenic mice expressing ChR2 (Channelrhodopsine-2) in inhibitory neuronal populations [14]. With this technique, we found that optogenetic activation of inhibitory neurons in anesthetized and freely moving mice strongly affects the control of movements. Specifically, we show that the organization of the inhibitory circuit in the spinal cord for the control of movement is arranged in a descending fashion with inhibitory centers located more rostral than the targeted motorneuron pool, which innervate the specific muscles. In marked contrast to their influences on the motor behavior, those higher inhibitory centers have minimal effect on the transmission of sensory and proprioceptive information to the cortex.

## Results

### Inhibition of motor outputs evoked by intraspinal stimulation

**Figure 1:**
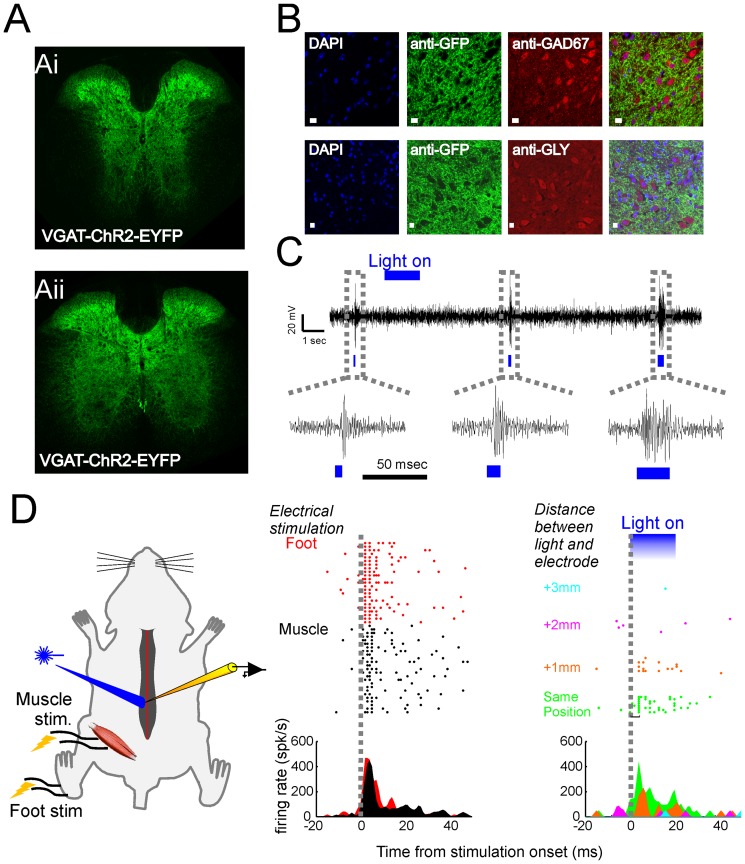
Characterization of ChR2-EYFP expression in VGAT-ChR2(H134R)-EYFP line 8 BAC transgenic mice. (**A**) Transverse sections of the spinal cord at the thoracic (Ai) and lumbar level (Aii). In green endogenous GFP expression, which is similarly distributed both at the thoracic and lumbar spinal levels. (**B**) Immunostaining and co-localization. First column: DAPI staining; Second column: anti-GFP staining; Third column: anti-GAD67 staining (first row) or anti-GLY staining (second row); Last column: merged image of DAPI, anti-GFP and anti-GAD67/anti-GLY staining. Scale bar: 10 µm. (**C**) Multiunit responses upon light stimulation of the spinal cord. (**D**) Identified single neuron. Single neuron responses in the spinal cord were evoked both by stimulation of the muscle, of the foot and by optically stimulating the same area of the spinal cord where the electrode was inserted. As soon as the light was moved away from the electrode (color coded) - with a distance greater than 1 mm - the response to the light stimulation was no longer present.

In order to access to the widest population of inhibitory interneurons in the spinal cord, we used VGAT-ChR2 [14] mice which express ChR2 in neurons –both GABA and Glycine [15], [16] populations - under the vesicular γ-aminobutyric acid (GABA) transporter (VGAT) (ChR2 gene fused to the Enhanced Yellow Fluorescent Protein-EYFP and to VGAT or Slc32a1 promoter). High expression of ChR2-EYFP was present over the entire spinal cord (see Fig. 1A). As previously shown for the cortex [14], co-immunostaining of GAD67, GLY and EYFP (see Fig. 1A–B; ChR2 positive neurons are identified by the presence of EYFP fluorescence on the cell membrane) showed clear co-localization throughout the spinal cord (GLY+ included in the set of EYFP+ neurons: 46% in the dorsal, 80% in the middle and 84% in the ventral sector; GAD67+ included in the set of EYFP+ neurons: 98% in the dorsal, 83% in the middle and 50% in the ventral sector, n = 4 mice, 2/3 sections per mouse; many of those neurons – which are not estimated - co-express Glycine and Gaba, see [16], [17], [18]). These results suggest that ChR2-EYFP expressing neurons in this line are indeed inhibitory interneurons. Extracellular single unit recording *in-vivo* confirmed that blue light photostimulation of the spinal cord was able to trigger the activity of single neurons (Fig. 1C). Figure 1D shows an example of responses of an identified single neuron and its responses when blue light was applied over the spinal cord. As soon as the light was moved away from the recording electrode, the evoked response disappeared.

**Figure 2:**
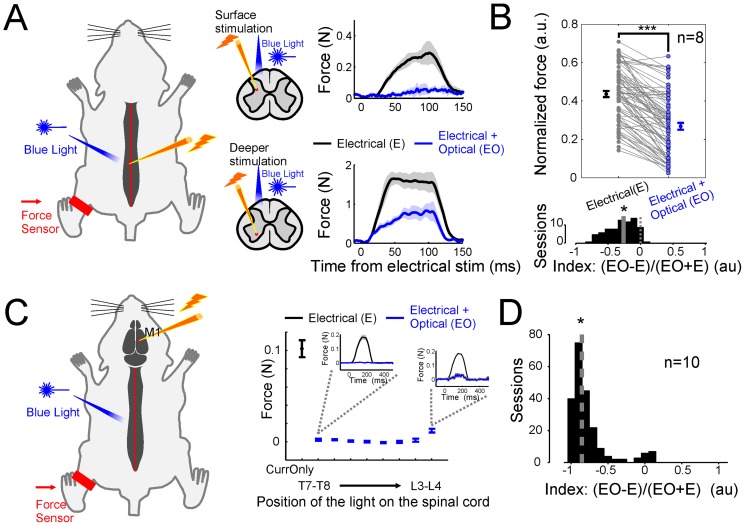
Motor output evoked by intraspinal/cortical stimulation. (**A**) Motor output evoked by intraspinal stimulation of anaesthetized mice. Isometric forces were recorded at the ipsilateral ankle of the side of intraspinal stimulation. Blue light was shone at the same location where electrodes were inserted. Traces of the force evoked by intraspinal stimulation at different depths are shown without (black traces) and with (blue traces) coupling electrical and optical stimulation. (**B**) Difference of the normalized population of the evoked forces with and without electrical and optical stimulation coupled together (p<0.05, Wilcoxon sign-rank test, n = 8 animals). An index (see Methods, lower subpanel) quantified the amount of reduction (p<0.05, Wilcoxon sign-rank test). (**C**) Motor output evoked by cortical stimulation of anaesthetized mice. Isometric forces were recorded at the contralateral ankle. Blue light was shone through an optical fiber, which was moved along the rostro-caudal axis of the spinal cord. On the right the average force evoked by cortical stimulation (in black - current only) and coupling electrical and optical stimulation (in blue) at different positions of the spinal cord (see also Fig. 3). In the insets it is shown the complete time profile of the force from which the averages were calculated. (**D**) The index quantifies the overall amount of the reduction (p<0.05, Wilcoxon sign-rank test, n = 10 animals).

In the first experiment, we probed the inhibitory effect of optical activation of VGAT-ChR2 neurons in anesthetized animals by evoking movements by intraspinal electrical stimulation of the spinal cord and by monitoring the produced hindlimb isometric force by means of a force sensor attached to the ipsilateral ankle (see Fig. 2A and Methods). We paired intraspinal electrical stimulation with a non-invasive light stimulation delivered at the same location through an optical fiber (see Methods). When electrical stimulation was coupled with light stimulation, the magnitude of the endpoint forced produced was strongly reduced (Fig. 2A). The amount of reduction depended on the depth at which the electrical stimulation was applied (Fig. 2A, index vs depth r^2^ = 0.33, p<0.05). Across all depths, the reduction of the force evoked by coupling the optical with the electrical stimulation (Fig. 1D) was on average of about 50% of the total force evoked by the electrical stimulation alone (median reduction 43%, n = 8 mice, p<0.05 Wilcoxon sign-rank test).

### Inhibition of motor output evoked by cortical stimulation

**Figure 3:**
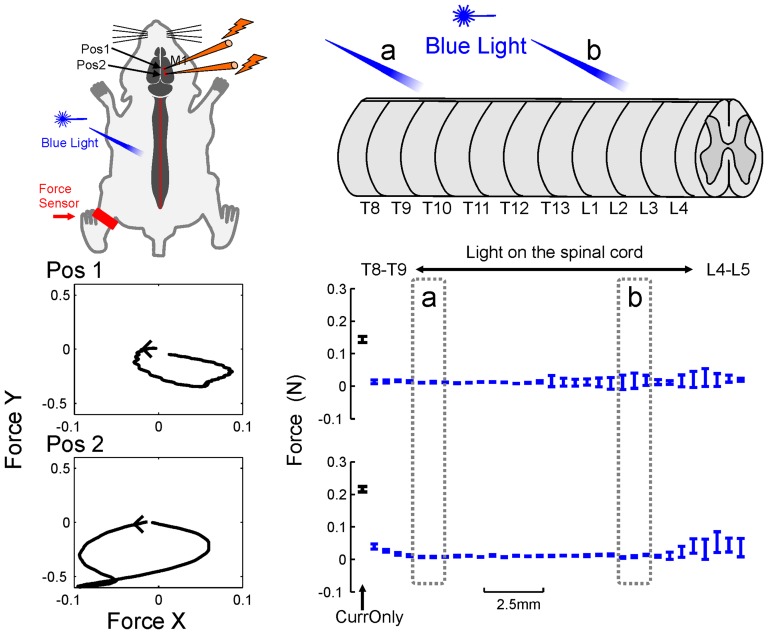
Electrical stimulation at multiple cortical sites with simultaneous optical stimulation of the spinal cord. Stimulating two different positions of the motor cortex evoked two different movements. Force x/y provide the time profile of the force on the x/y axes of the force sensor: x represents direction of the movement towards the head/back and y movements towards the tail of the animal. Electrical stimulation was combined to light stimulation of different sectors of the spinal cord with 500 µm resolution. In (a) light was shined approximately at the level of T9–T10 and it produced a complete suppression of the module of the force independently on the type of movement evoked. In (b) light was shined approximately at the level of L2–L3 and it produced a weaker and more variable suppression of the two evoked movements.

In the same anesthetized preparation of the previous experiment, we evoked movements by electrically stimulating the motor cortex (Fig. 2C). We combined the electrical stimulation of the contralateral motor cortex with light stimulation of the (ipsilateral) spinal cord. In this case, light was shone at different levels of the spinal cord (from middle thoracic to upper/middle lumbar with about 0.5–2 mm steps, see Methods) by means of a movable optical fiber. Like previous observations, the end-point force produced by electrical stimulation of the motor cortex was strongly reduced when the electrical and light stimulation were coupled (Fig. 2C). Interestingly, independently on the specific direction/pattern of hindlimb movements, light stimulation of the middle-lower thoracic sector had the strongest suppression of all the hindlimb movements evoked by cortical stimulation (Fig. 2C–D and Fig. 3, median = −0.85, p<0.05, Wilcoxon sign-rank test, n = 10 mice), which depended upon the activation of motorneurons located in the middle lower lumbar level of the spinal cord. Taken together, these two experiments illustrate that optogenetic stimulation of inhibitory interneurons effectively suppressed movements and furthermore, the strongest and more general inhibitory effects were mostly present at the middle-lower thoracic level and they propagated in a caudal manner. Also, suppression of cortically evoked movements was contingent upon activation of the spinal cord with blue light, which drives ChR2 neurons. Indeed, light of a different wavelength i.e. green, was not able to suppress the evoked movement (see Fig. S1A). Furthermore, because in anaesthetized animals there is no tonic muscle activity, blue light alone shined on the spinal cord of VGAT-ChR2 mice was not able to produce any movement (see Fig. S1B).

### Inhibition of movements in freely moving animals

**Figure 4:**
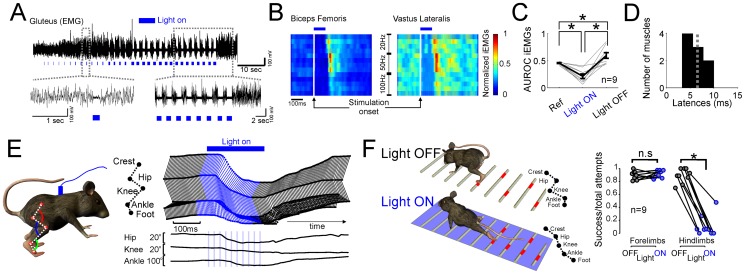
Activation of inhibitory neuron in awake freely moving animals. (**A**) EMG recordings from the gluteus muscle with and without light stimulation. (**B**) Normalized (to the peak activity) EMG of simultaneously recorded agonist and antagonist muscles (see also Fig. S3). Spinal cord was stimulated for 100 ms with pulses of 5 ms duration and different frequencies. Similar suppression of the muscle responses were observed independently from the parameters of the stimulation used. (**C**) Population measure of the AUROC iEMG before (Ref), during (Light ON) and after (Light OFF) stimulation. During the stimulation, muscle activity was suppressed, but right after the stimulation ended a rebound of over activation of the muscles was observed (AUROC iEMGs, asterisks indicate significant difference p<0.05, Wilcoxon sign-rank test corrected for multiple comparisons). (**D**) Population measure of the latency of the suppression (median 6.5 ms from stimulation onset). (**E**) Kinematic reconstruction of a movement recorded with an high-speed camera. The animal loose the balance after about 30 ms from stimulation onset. See Movie S1. (**F**) Ladder skilled test. The animal was placed in a ladder apparatus where it walked by correctly placing the paws on the rungs of the ladder. In the absence of light stimulation, the animal correctly alternated its limbs. Nevertheless, when light was shone on the spinal cord, the ipsilateral limb was blocked from moving (p<0.05, Wilcoxon sign-rank test), affecting his ability to walk on the rungs (see Movie S2). Optical fiber was implanted at T12 or higher.

**Figure 5:**
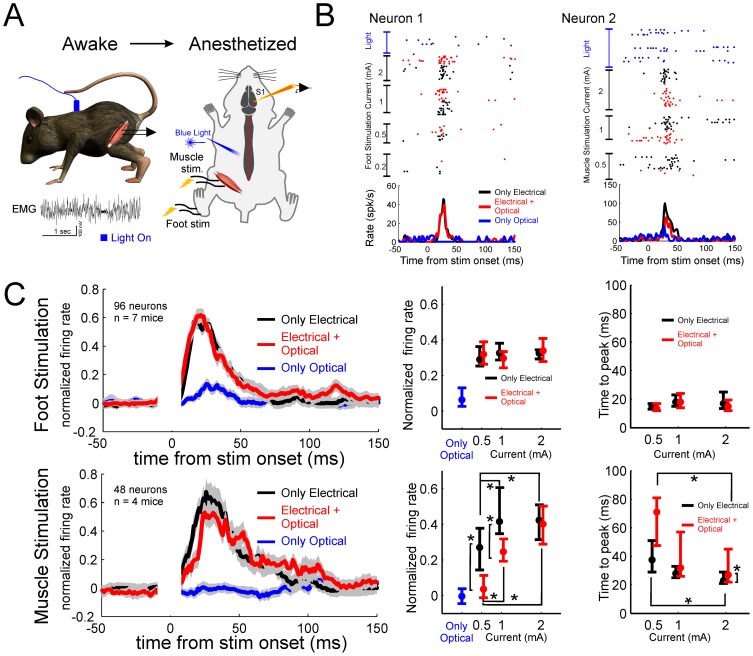
Sensory recording during peripheral stimulation. (**A**) First, muscle(s) suppressed during the awake stimulation of the previous experiment (Fig. 4) were identified. Then, the animal was anesthetized and single neurons were recorded in the sensory cortex. Activity was evoked by electrical stimulation of one of the affected muscles and of the foot. (**B**) Example of a two single neurons responding to the stimulation of the foot (Neuron 1) and of the muscle (Neuron 2) with different intensities of the current. Light stimulation is presented as well. (**C**) Population activities in response to foot and muscle stimulation. On the left, normalized population activities obtained at 2 mA. In the center, average normalized activities to the peak activity. On the right, time to peak activities. Asterisks indicate significant differences (from 10 ms to 70 ms after stimulation onset) either between current intensities or between stimulation conditions (two separate – Only Electrical and Electrical + Optical - Kruskal-Wallis p<0.05 post-hoc Bonferroni corrected for multiple comparisons with factor intensities, and Wilcoxon sign-rank test p<0.05 between Only Electrical vs. Electrical + Optical conditions).

To assess the behavioral effect of inhibitory interneurons, we optogenetically activated spinal interneurons in awake freely moving animals. No previous study has performed optogenetic manipulation of spinal circuits in awake, freely moving animals (but see Alilain et al [19] for an example of optical stimulation of the spinal cord in vivo). We implanted an optical fiber by means of a spinal adapter (see Methods) - in the middle/lower portion of the spinal cord targeting T10, where in the previous experiment we found the strongest global inhibitory effect. Brief period of optical stimulation (50–100 ms, 5 ms pulse 20–50–100 Hz frequency) of the spinal cord produced a loss of the muscular tone during both locomotion and stance. The effect was quantified by means of EMG recordings (Fig. 4A–D) and analysis of the behavior/kinematic (Fig. 4E–F). EMG recordings revealed a complete suppression of the muscle activity during light stimulation (Fig. 4A) for all muscles - agonist and antagonist - caudal to the stimulation point (Fig. 4A–D and Fig. S3, latency median = 6.55 ms, EMG reduction quantified by means of Friedman test with factors rest, Light ON, Light OFF, chi-squared = 18, p<0.05, post-hoc test: p<0.05 Wilcoxon sign-rank test, Bonferroni corrected). All tested frequencies were able to produce a suppression of the muscle activity. However, stimulation at 50 Hz produced the maximum suppression (Kruskal-Wallis with 3 conditions – 20, 50, 100 Hz-, p<0.05, Bonferroni corrected). Furthermore, after the stimulation ended, we observed a rebound over-activation of the muscles caudal to the stimulation point (Fig. 5C, light OFF vs rest, p<0.05 Wilcoxon sign-rank test, Bonferroni corrected). Such rebound effect, happening 17.5 ms (median value) after the end of the light stimulation - stronger for 50 Hz with respect to 100 Hz stimulation (p<0.05, U-test) - could well be necessary to compensate for the loss of muscle tone and to regain correct walking patterns (Fig. 4E, see Movie S1). It has to be noticed that suppression and recovery of muscle activity were very fast (i.e., suppression 6.55 ms vs. recovery 17.5 ms), and frequencies higher than 20 Hz were necessary to guarantee a continuous suppression of the movement. Next, we tested the behavioral consequences of prolonged light activation of inhibitory neurons. On a skilled (ladder test) locomotor task (Fig. 4F and Movie S2) in absence of stimulation, the animal walked properly with a correct alternation of limbs. However, when blue light was shone over the spinal cord (duration >500 ms, 5 ms pulse 50–100 Hz frequency), the movements were suppressed or strongly compromised (correct placement light ON vs. light OFF, forelimb p >0.05, hindlimb p<0.05, Wilcoxon sign-rank test, n = 9 animals, Fig. 5F) often resulting in the dragging of the affected hindlimb (see Movie S2). As soon as the light was turned off, the animal immediately recovered stance and normal walking behavior.

### Sensory consequences of the inhibition of movements

In a subset of the animals tested in the previous experiment, we evaluated the sensory consequences of the inhibition of the motor behavior. Once we identified the muscle(s) suppressed during light stimulation (Fig. 5A), we anesthetized the animal and we recorded single neuron activity (see Methods) in the somatosensory cortex evoked by electrical stimulation of one of the muscles affected. At the same time, we tested the activity in sensory-cortical neurons evoked by stimulation of the hind-paw/foot (i.e., sensory and nociceptive fibers) [20]. Overall, the cortical sensory responses evoked by peripheral electrical stimulation showed the same time profile with both the electrical stimulation alone and the electrical coupled to the optical stimulation of the spinal cord (see an example in Fig. 5B). More specifically, the time profile in both conditions was the same (Fig. 5C). In the case of foot stimulation, no difference was found in either average or peak responses (Fig. 5C) at any level of stimulation (p>0.05, Wilcoxon sign-rank test). In the case of proprioception (muscle) stimulation, an effect of the light was found only for low currents (Fig. 5C). For higher intensity of the current, the average activations were similar. Taken together these results suggest that projection to the cortex of proprioceptive and sensory information at the paw were marginally perturbed by the light stimulation.

### Consequences of activation of inhibitory neurons on reflexes

**Figure 6:**
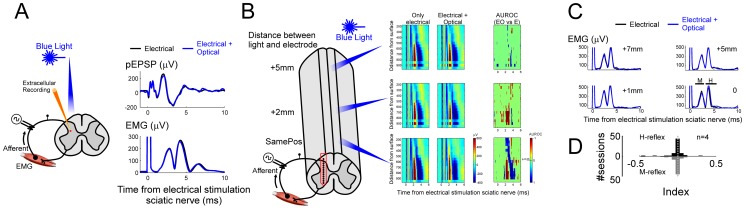
Effect of sensory and optical stimulation on M-/H-reflex and population excitatory post-synaptic potential (pEPSP). (**A**) Schematics of the experimental set-up. Extracellular recordings were performed during stimulation of the sciatic nerve. Electrical stimulation of the sciatic nerve evoked both a population EPSP in the spinal cord and a M-/H- reflexes in the muscle. (**B**) pEPSPs were recorded at different depths by means of a linear array (see Methods). On the left, it is shown the position of stimulation of the spinal cord with respect to the recording electrodes (reported as rows on the right). On the right, the three columns represent respectively the effect of the pEPSP evoked by electrical stimulation (of the sciatic nerve), by combining electrical and optical stimulation, and AUROC indices (see Methods) to quantify the differences with vs. without optical activation. Population potentials at different depths were mostly perturbed when light was applied at the same position and marginally when it was in the proximity (about 2 mm) of the recording electrode. No difference was present when the light was shined at greater distances from the recording electrode. (**C**) Electromyography activity evoked by combining electrical stimulation of the sciatic nerve with (blue) and without (black) optical stimulation at different position of the spinal cord. Zero distance indicates that light was shone at the same position of the recording electrodes. No effect of the light stimulation on neither M- nor H- reflexes (p>0.05, sign-rank test) was observed independently on the position of the light.

In the previous experiments we showed that cortically evoked and voluntary movements can be inhibited with optical activation of inhibitory neurons at the thoracic level while sensory/proprioceptive information can still reach the cortex. We wonder about the fate of the spinal local processing of proprioceptive signals at the lumbar level while activating inhibitory neurons at the thoracic level. In order to test the transmission of sensory information inside the spinal cord we electrically evoked reflexes: in an anesthetized animal (see Methods), we stimulated the sciatic nerve and we simultaneously recorded muscle activations from distal muscles (tibialis anterioris, gastrocnemius and foot-pad muscles) together with intraspinal population excitatory post-synaptic potential (pESPS, see Fig. 6A).

Electrical stimulation of the sciatic nerve evoked potentials at the lumbar spinal cord level and, at the same time, produced muscle reflexes (M and H, see Fig. 6A). Population excitatory post-synaptic potentials were recorded at the middle lumbar level (L3–L5) of the spinal cord by means of a linear array which allowed a simultaneous sampling of the potentials at different depths (see Methods). Differences in the evoked pESPS with and without optical stimulation were quantified by means of an AUROC index (see Methods), which assume values between 0 and 1 (Fig. 6B). A value around 0.5 indicates no difference between conditions while a value of 1 (greater) or 0 (lesser) indicates a perfect discrimination between the potential evoked by electrical or electrical coupled with optical stimulation. We observed a change on the population potentials only when the light was shined on the lumbar region. These differences consisted in an overall reduction of the potentials at every depth. Interestingly, the difference between potentials decreased and finally disappeared as soon as the light was moved more rostrally from lumbar towards thoracic level. At the same time, we found that both M-reflex and H-reflex (see Fig. 6C) were not affected by combining light stimulation of the spinal cord with electrical stimulation of the nerve at all spinal levels: reflex-evoked muscle activity with vs. without light (Fig. 6D) were not different (p>0.05, Wilcoxon sign-rank test, n = 4 mice).

Although we did not find effects of suppression of reflexes at the intensities of this study, it has to be noted that optogenetic activation of the lumbar spinal segment can suppress the H-reflex evoked at low current intensities (see Fig. S2). This effect might be the consequence of a stronger suppression of the monosynaptic fiber from muscle spindles rather than rostro-caudal suppression as observed for the pEPSP (Fig. 6B).

## Discussion

We showed that the VGAT-ChR2 mouse is a suitable model to study the role of inhibitory interneurons in shaping spinal cord modulation of motor output. Although activation of spinal inhibitory interneurons affected movements, the processing of peripheral sensory information was minimally affected. Furthermore, we showed that the spinal inhibitory interneurons exert inhibitory effects only to motor-output circuitries located caudal to themselves.

The use of light to activate neuronal populations of the spinal cord is a relatively new technique. In this regard, the influence of confounding effect e.g. heating, cannot a-priori be excluded. We minimized the heating both by keeping the tip of the optical fiber at a certain distance and by applying agar solution on the surface of the spinal cord. Furthermore, heating itself may activate pain fibers which run in the dorsal spinal cord. We did not observe any pain-related responses such as hunched posture, grooming of the implant, or twitching during stimulation of the spinal cord in awake freely moving animals. Typically, those type of responses were observed in control subjects for much higher stimulation intensities. Also, as control we performed the same experiments in wild type animals and/or using laser tuned to a different wavelength (green). It is also very unlikely that the spread of the light might have activated neurons far from the site of stimulation since single neuron activity was not evoked when the optical fiber was placed more than 1 mm away from the recording electrode (see Fig. 1C).

Taking into consideration the fact that sensory afferent fibers from hind-limb proprioceptors ascend to the L1 segment but rarely more rostrally [21], several lines of evidence in our experiments point toward a purely rostro-caudal inhibition in addition to the local modulation of sensory information [22]. First, all movements evoked by electrical stimulation of the cortex in the anesthetized (Fig. 2–3) and awake (Fig. 4) preparations were completely suppressed when the optical fiber was placed above T12. Second, effect of light stimulation on the population excitatory post-synaptic potential (pESPS) was only local (Fig. 6B) suggesting that thoracic stimulation did not affect the pESPS of the hindlimbs. Third, reflexes were not affected by light stimulation (Fig. 5C–D). Fourth, in the awake preparation the effect of stimulation of the thoracic spinal cord showed inhibition only of the hindlimbs while forelimbs were never affected (Fig. 4F).

The spinal cord has been shown to be organized according to function [6], [23]. For example, motor neurons are clustered into bundles which correspond to specific muscles [6]. Furthermore, evidence suggests that neurons activated by glutamatergic agonist in the dorsal sector of the spinal cord have a modular organization that corresponds to distinct motor synergies [7], [8]. Also, gradients of excitability have been documented for the activation of the central pattern generator for locomotion at the lumbar and sacral levels of the spinal cord [3], [24], [25], [26], [27], [28], [29]. We show here that unlike the excitatory spinal circuits, some inhibitory circuits for the control of hindlimb movements exhibit a strictly descending influence similar to the effect shown in *ex*-vivo studies on neonatal rodents [12], in the breathing/swallowing pattern generator of decerebrated cats [13] and in spinal cord of injured subjects [30], [31]. We can offer three possible explanation of how such an effect can take place. First, inhibition can indirectly affect motor-neurons by inhibiting their excitatory input through a downstream long range proprio-spinal inhibition which has been shown to extend from cervical to lumbar/sacral segments [32], [33]. Second, inhibitory neurons at the thoracic level can be a class of premotor interneurons. In fact, Tripodi et al. [9] and more recently Levine et al. [11], reported that premotor interneurons monosynaptically connected to motor neurons in the middle/lower lumbar levels reach the lower thoracic spinal segments (and, presumably, above that level, see also [34]). As Levine et al. [11] described many of this neurons are inhibitory. Third, rostro-caudal inhibition can be produced by inhibiting descending excitatory pathways [35]. At the current state, we cannot discern if we are activating one or multiple of these circuits because the evidence of descending inhibition presented here relies on an unspecific expression of ChR2 via the VGAT transporter gene, thus preventing us from identifying the contributions of specific classes of inhibitory neurons (see Fig. 1B). Further studies will be needed to understand if a specific or several classes of interneurons are involved in the observed descending inhibition and their functional circuit.

What is the function of this rostro-caudal inhibition? One possibility, given the global influence of the observed inhibition, is that these neurons might be involved – along with other mechanisms - in implementing the atonia that has been described during rapid eye movement (REM) sleep. Supporting this idea, recent experiments have shown an increase of the level of GABA/glycine in the spinal cord during REM sleep [36]. Furthermore, inhibitory interneurons in the spinal cord are the target of excitatory descending fibers from the sublaterodorsal nucleus which is involved in the control of REM sleep [37]. Indeed, deletion of VGAT in the spinal cord resulted in twitching and jerking movements during REM sleep [38]. Another possibility is the involvement of inhibitory neurons in the control of posture and locomotion. In fact, inhibition is a critical component for the control of movement to balance and to coordinate the effect of excitation [39], [40], [41]. For example, recently it has been shown that inhibitory interneurons are the fundamental elements of the limb central pattern generator (CPG) that coordinate flexor-extensor motor activity [42], [43]. Also, inhibitory neurons acts on the CPG for the regulation of the speed of locomotor step cycle [1], left-right alternation [44], and crossed muscle coordination [3].

The rostrocaudal lateralized inhibition and timing of activation we observed point more towards an global inhibition of caudal motor neurons by means of premotor circuits [11]. In this respect, one of the functions of the rostro-caudal inhibition to the control of locomotion can be as a negative gain for force control on the muscles activation [21]. Another, non-mutually exclusive explanation can be the inhibitory control of synergistic muscles which might represent a more efficient way to control movements [23]. Indeed, as shown by Levine et al [30] premotor synergistic neurons are in great number inhibitory. They can have direct control of motorneuron activities or also they can control the activity of other premotor-synergistic neurons. In such a fashion, the rostro-caudal inhibition might represent a more efficient way to control and to combine synergistic activations.

In conclusion, in this paper we presented a new approach for investigating the spinal inhibitory network in both anesthetized and awake freely moving mice by means of optogenetics. These experiments showed a new rostrocaudal inhibitory effect at the middle/lower thoracic level in addition to the well-established local organization for postural control and locomotion at the lumbar and sacral levels.

## Materials and Methods

### Subjects and surgical methods

VGAT-ChR2 mice (donated by Guoping Feng at MIT) were used in this study. The line of mice used is commercially available from Jackson Laboratories (strain name: B6.Cg-Tg(Slc32a1-COP4*H134R/EYFP)8Gfng/J). All procedures were approved by the Committee for Animal Care of MIT (protocol number 0910-080-13). Animal care was in accordance with guidelines from the National Institutes of Health and approved in advance by Committee for Animal Care of MIT. Surgical procedures were performed either under isoflurane or ketamine/xylazine anesthesia and all necessary precautions were taken to minimize animal suffering.

### Spinal cord preparation

Mice were anesthetized with 2–3% isoflurane in 80% oxygen and then maintained on 1–2% isoflurane in 80% oxygen. Body temperature was maintained at 37°C with a heating blanket (Harvard Apparatus) and heating lamps. Dorsal surface of the thoracic spine was shaved and cleaned (three alternating washes of 70% ethanol and povidone-iodine). Following a midline incision, soft tissue was retracted to expose the lateral masses of the thoracic and lumbar vertebrae scraped the bone clean on the top and the sides. We severed tendons attached to the vertebrae using surgical scissors and clamped the vertebras into a stereotactic frame. Laminas were removed by means of spring scissors and spinal landmarks (main vessels) identified. Excess bleeding was stopped by means of cotton swabs and gel foam. Then, the exposed spinal cord was covered with a thin layer of 2% agarose in PBS in order to keep the spinal cord moist and to reduce the heating produced by light stimulation.

### Spinal implant

The same procedure described above to expose the spinal cord was implemented to expose a much smaller region of the spinal cord composed of 1–2 vertebrae in the middle/lower thoracic region (the target level of the spinal cord was about at T10). Spinal implant was a custom-made modified (smaller) version of the one reported in Farrar, et al. [45]. Surgical implantation followed the same procedure reported in the paper by Farrar et al. In summary, small metal bars clamped the vertebras and they were kept in place by screwing a plate on them. The plate had an aperture to access the spinal cord that was filled with Kwik-Sil silicone elastomer (World Precision Instruments). A cannula, maintained with a cannula holder on a stereotactic frame, was slowly lowered on the spinal cord 200–400 µm lateral from the midline. The cannula was then secured by means of cyanoacrylate glue and dental acrylic and covered with a dust cap. The skin was secured with vetbond and sutures. The animal was allowed to recover from anesthesia with the help of heating pads and was returned to the cage once it showed regular breathing and locomotion. Mice were single housed post-surgery and throughout the rest of the experiments.

### Head fixation

Following an incision along the midline of the skull, the fascia and fat overlying the skull surface were scraped away. Subsequently, a metal head-plate was attached to the skull with cyanoacrylate glue and dental acrylic. A 2×2 mm craniotomy was made over the hindlimb area of the primary sensory (S1) and motor cortex (M1) that was later covered with a thin layer of 2% agarose in PBS. Head plate was then screwed into a stereotactic frame.

### EMG/cuff electrodes implantation

Mice were anesthetized with isoflurane (2%) and placed on a heating blanket. Supplemental does of isoflurane (1–2%) were administered as needed. Skin was disinfected with three alternated application of 70% ethanol and Povidone-iodine. Two to three small skin incisions were made on the hindlimb over target muscles and along the back. EMG wires, already attached to the connector, were braided and tunneled under the skin. Each target muscle was implanted with a sterile pair of teflon-coated stainless steel wires (A-M system 795500) inserted into the muscle belly by means of a needle. The connector was attached to the spinal implant by means of cyanoacrylate glue and dental acrylic. In order to check the correct location of the electrodes, brief pulses of current were passed through the wires to stimulate the muscle. For recording the muscle activity, the signal was amplified and filtered (5–1000 Hz), using an A-M Systems (Sequim, WA, USA) Model 1800 AC amplifier. The same procedure was used to implant a handmade cuff electrode around the sciatic nerve.

### Electrical Stimulation and recording

Microelectrodes (stainless steel/tungsten, tip diameter 0.5–1 µm, 2–5 MOhm) or arrays (NeuroNexus multi-site electrode - A1-X16-3 mm-50-413) were inserted into the spinal cord or into the sensory-motor cortex under visual inspection through a microscope. For stimulation, we applied biphasic trains of negative current stimulation (intra-spinal: 1–15 µA, 100–200 ms train, 100 Hz, 0.1–0.2 ms pulse; cortex: 50–300 µA, 100–200 ms train, 200–300 Hz, 0.1–0.2 ms pulse) through those electrodes (BAK stimulator). Sites between 300–400 µm lateral to the cord midline were examined with different depth. For the stimulation of the motor cortex, we started with the stereotaxic coordinate (M1) and moved the electrode until the area corresponding to the hindlimbs was identified. Currents were adapted to the site/depth as the minimum current necessary to observe a muscle twitch and/or a movement detectable from the force sensor. Sensory stimulation was evoked by means of biphasic single pulses (0.2–8 mA) delivered either to the foot by needle electrodes or to the electrodes implanted into the muscles. Recorded signals were amplified by a 1700 AC Amplifier or by a 3000 AC amplifier (A-M system).

In the anesthetized preparation (ketamine/xylazine mix), the isometric force of the hindlimb evoked by microstimulation was measured by means of a six-axis force transducer (ATI sensor, sampled at 20 kHz) using a cuff wrapped securely around the ankle of the animal. The transducer was mounted on a positioning device that allowed the ankle to be fixed in any position within the workspace of the hindlimb. We generally only report the results based on an analysis of the module of the force evoked. The background resting force measured prior to stimulation was subtracted from all force measurements and the remaining active evoked force was used for all subsequent analyses.

### Optical Stimulation

We employed a diode-pumped solid state blue laser (473 nm, maximum power 100/200 mW, Laser Century) or green laser (532 nm, maximum power 200 mW, Laser Century) coupled via FC/PC terminal to a 200 µm core optical fiber (ThorLabs). Tip of the optical fiber was placed just above the spinal cord (200–300 µm) by means of a movable device. In simultaneous intra-spinal electrical stimulation and optical stimulation, the optical fiber was placed with an angle of about 30 degrees at the same location of the electrode. For awake preparation, 100 µm core fiber (ThorLabs) was attached to a ceramic ferrule (1.25 mm outer diameter - precision fiber products). Typically, light pulses of 0.5–2 mW in anesthetized and 1–4 mW in awake preparation were utilized. For each session we established the minimum power to observe a suppression of the evoked/spontaneous movement or we used 2 mW (for anesthetized)/4 mW (awake preparation) in case we could not make preliminary observations. Once fixed, we maintained the same power for the whole session. Time of stimulation and parameters (frequency, duration pulses and duration of the stimulation) were controlled by means of custom made Labview scripts. Because of the out-of-spine optical stimulation, the surface of the spinal cord and the dura might produce different scattering responses when light is shined on the spinal cord. Also, in the awake preparation we found often an opaque connective tissue on the surface of the spinal cord and under the optical fiber. Hence, we could not use exactly the same parameters across animals and it is hard to establish exactly the depth of penetration of the light. Nevertheless, we expect that because of the very low intensity of the light utilized to obtain a behavioral effect, the suppression of the movement should have been produced already with the activation of the middle-upper sector of the spinal cord. Furthermore, we kept the optical fiber at a distance of 300–400 µm from the midline. If we assume a spherical distribution of the intensity, the stimulation in depth on one side will affect also the other side of the spinal cord that would have produced in a bilateral inhibition in the awake preparation, which was rarely observed.

### Index of electrical and electrical-coupled-optical stimulation

We quantified the effect of the force and absolute EMG produced by electrical stimulation alone and electrical-coupled-optical stimulation by means of a selectivity index calculated as the difference between the force/absolute EMG evoked by electrical-coupled-optical stimulation and the force/absolute EMG evoked by electrical stimulation alone then normalized by their sum.

### Data analysis

Data were stored at 20 kHz on a Datawave logger and analyzed offline using custom software written in Matlab (Mathworks Inc.). Spike sorting was offline performed by PCA and template analysis of the waveforms (Datawave). Spike trains were aligned either to the electrical stimulation (both when it was performed in isolation or when it was coupled with the optical stimulation) or to the optical stimulation (when it was performed in isolation). Period of time before and after 10 ms from the electrical events were excluded from the analysis because, in some cases, compromised by stimulation artifacts. In order to establish if a neuron was responding to the sensory stimulation we run a paired running Wilcoxon sign-rank test for non-overlapping bins of 10 ms (for the first 150 ms after stimulation onset) and we compared them with baseline (from 150 ms to 50 ms before stimulation onset). Whenever a neuron showed a significant response in one of the bins (p<0.05 corrected for the number of bins), it was considered as sensory related response. We then normalized the firing rate by dividing the net activity (subtracted the baseline) by the peak activity.

The EMG activity was band passed between 10 Hz and 1 kHz, rectified, and smoothed (5-point average with a span of 10 ms).

### AUROC analysis

We used an Area Under the Receive Operating Curve (AUROC) index to evaluate the difference between the EMG pattern before and after optical stimulation. AUROC values were evaluated by comparing the EMG activity in a sliding window of 5 ms with a step of 0.5 ms from 200 ms before to 500 ms after optical stimulation with respect to a baseline activity (activity in the 200 ms interval before optical stimulation). The same index was used to compare the pESPS evoked by the electrical stimulation alone and by the electrical stimulation coupled with optical stimulation (step 0.05 ms, window 0.25 ms).

### Treadmill and analysis of the kinematic

A custom made treadmill was adopted in order to study the animal locomotion. Skin marks or reflective markers were placed on the hindlimb and movements were monitored with high-speed monochromatic cameras (GigE 120 frame per second or Phantom 200 frame per seconds – gently provided by the Edgerton Center at MIT). Tracking and reconstruction of hindlimb movements were performed with kinovea software (http://www.kinovea.org/) and Matlab.

### Ladder skill test

For testing skilled locomotion a ladder was built similarly to the one proposed in Ghosh et al. [46]. The horizontal ladder consists of equally spaced (at 1-cm gaps) rungs. The ladder was 1 m long and elevated at about 40 cm. At least three trials with and without stimulation were video recorded over a 50 cm stretch. Videos were analyzed offline. When the paw was placed such that the limb did not slip from the rung (weight-supported steps), a step was noted as successful [46].

### Immunohistochemistry

Mice were perfused transcardially with cold PBS, followed by 4% paraformaldehyde (PFA) in PBS. Spinal cords were extracted and kept in 4% PFA at 4°C overnight and then left in sucrose (30%). Slices were collected at 20/40 micrometer of thickness in the cryostat. For immunostaining, each slice was placed in PBST (PBS + 0.2% Triton X-100) with 10% serum for 2 h at room temperature and then incubated with primary antibody at 4°C over night (mouse anti-GABA 1∶500, Abcam; chicken/rabbit anti-GFP 1∶500, Invitrogen; rat anti-glycine 1∶1000, ImmunoSolution). Slices then underwent three wash steps for 10 min each in PBST, followed by 2 h incubation with secondary antibody (1∶200 AlexaFlour 488 anti-chicken/rabbit, Invitrogen; 1∶200 AlexaFlour 546 anti-rat, Invitrogen; 1∶200 AlexaFlour 647 anti-mouse, Invitrogen). Slices were then incubated and mounted with DAPI (vectashield) on microscope slides and imaged using a confocal microscope (Zeiss LSM 5 Pascal Exciter). For cell counting, we analyzed (20× magnification) the dorsal, middle, and ventral regions of the lower thoracic spinal cord of 4 animals. ChR2-EYFP+/GLY+/GAD+ neurons were identified by means of co-localization of respectively the membrane/soma with DAPI.

## Supporting Information

Figure S1 Effects of electrical and optical stimulation on movements evoked by cortical stimulation. Animal preparation follows the one illustrated in the experiment 2 and Figure 2 of the main paper for cortically evoked movements with light shined over the thoracic level of the spinal cord. Optical stimulation was coupled with either a green laser (left panel, A) or a blue light (right panel, B).
(TIF)

Figure S2 M-/H- reflex evoked by electrical stimulation of the sciatic nerve at different intensities. EMG recordings were performed on the footpad of anaesthetized mice. On the left side of the figure EMG responses evoked by the stimulation of the sciatic nerve at different intensities of the current are shown in black (starting 1 ms after electrical stimulation events and not rectified). In blue are shown the traces obtained at the same intensities but coupling the electrical stimulation with optical stimulation at the spinal lumbar level. It is possible to observe a M-wave in the first 3–4 ms and the occurrence of the H-wave after about 5 ms. By increasing current intensities, the probability to evoke a spike increased. Nevertheless, only for low intensities it was possible to suppress the H-reflex. With higher intensities of the current the H-reflex was not suppressed. On the right side of the figure an index (see Methods) quantified the amount of reduction of the EMG activation by coupling electrical and optical stimulation both at lumbar and at the thoracic level. While with the optical stimulation of the lumbar spinal cord we observed a reduction of the H-reflex, we did not observe such an effect with optical stimulation of the thoracic spinal cord.
(TIF)

Figure S3 EMG and AUROC analysis in awake freely moving animals. Normalized (upper row) and AUROC EMG analysis (lower row) of simultaneously recorded muscles. Optical fiber implanted around T13-L1. Only the activity of the Gluteus was suppressed by the optical stimulation while the Vastus Lateralis was not affected. Same conventions as in Figure 5.
(TIF)

Movie S1 High speed recording of hindlimb movements. Skin markers identifying crest (red), hip (yellow), knee (green), ankle (blue) and foot (pink) were tracked in the high speed movie. Traces of the tracked movement are shown color coded. Movements were critically modified during period of light stimulation. Speed 50% of original.
(AVI)

Movie S2 Skilled ladder movements. The animal walked over the ladder by correctly placing the paws on the rugs of the ladder. During period of light stimulation only movements of the affected (hind) limb were compromised. Normal speed.
(AVI)
