# Supplementary figures and images for: Rostro-Caudal Inhibition of Hindlimb Movements in the Spinal Cord of Mice
Source: PLoS One. 2014 Jun 25;9(6):e100865. doi: 10.1371/journal.pone.0100865 (PMC4071039; doi:10.1371/journal.pone.0100865)

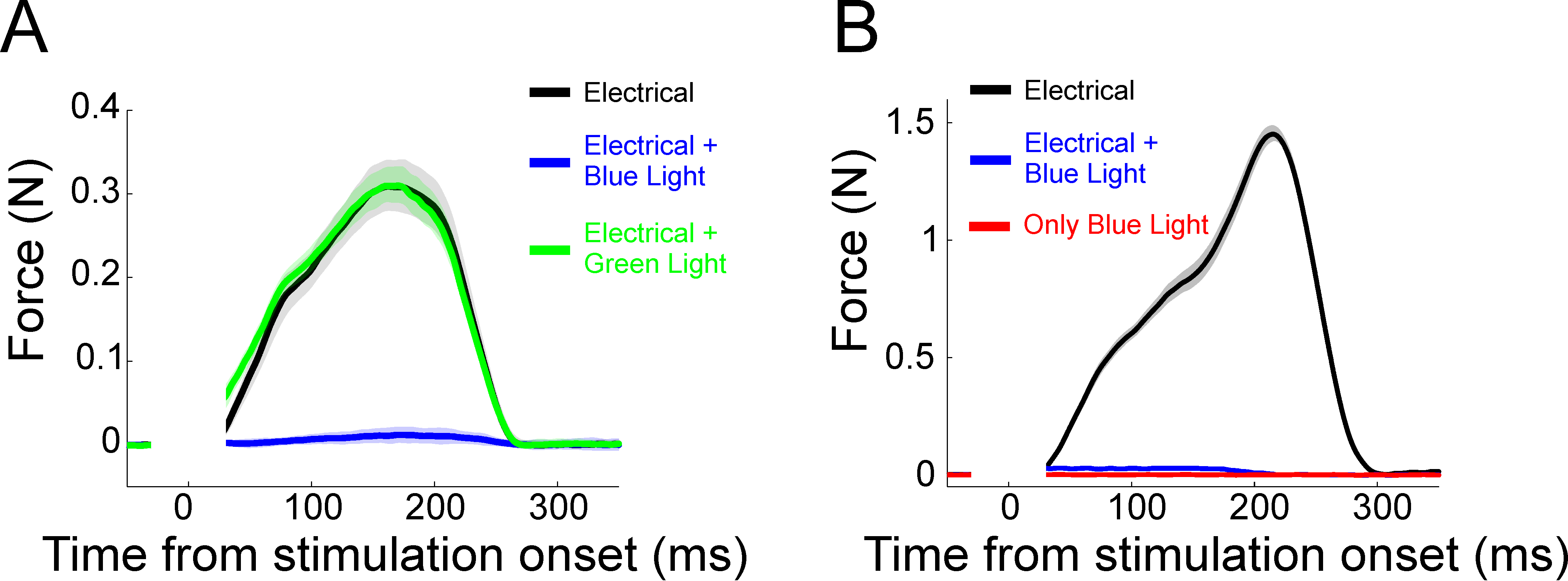

Supplement: Figure S1 — Effects of electrical and optical stimulation on movements evoked by cortical stimulation. Animal preparation follows the one illustrated in the experiment 2 and Figure 2 of the main paper for cortically evoked movements with light shined over the thoracic level of the spinal cord. Optical stimulation was coupled with either a green laser (left panel, A) or a blue light (right panel, B). (TIF) [file pone.0100865.s001.tif]

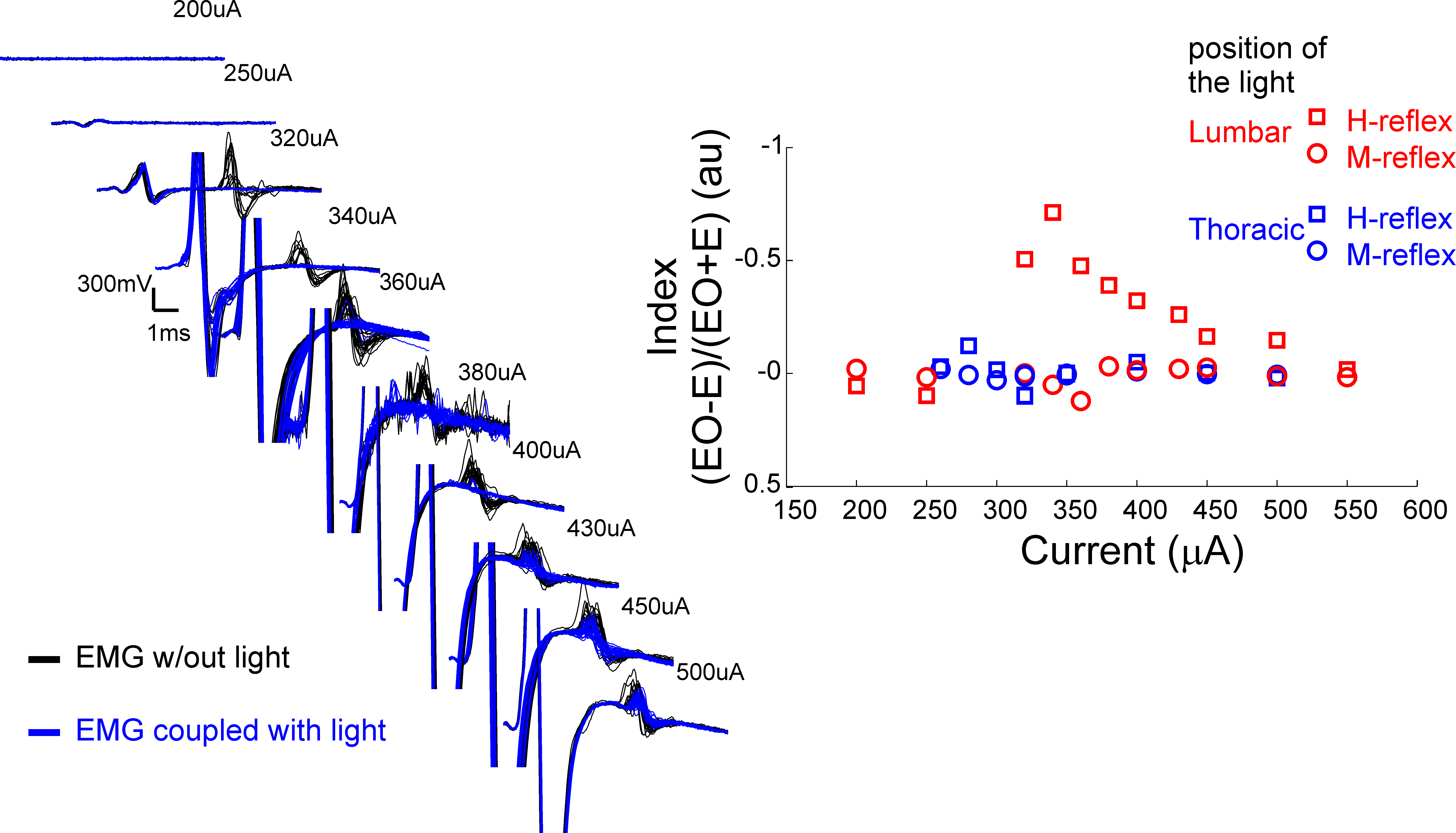

Supplement: Figure S2 — M-/H- reflex evoked by electrical stimulation of the sciatic nerve at different intensities. EMG recordings were performed on the footpad of anaesthetized mice. On the left side of the figure EMG responses evoked by the stimulation of the sciatic nerve at different intensities of the current are shown in black (starting 1 ms after electrical stimulation events and not rectified). In blue are shown the traces obtained at the same intensities but coupling the electrical stimulation with optical stimulation at the spinal lumbar level. It is possible to observe a M-wave in the first 3–4 ms and the occurrence of the H-wave after about 5 ms. By increasing current intensities, the probability to evoke a spike increased. Nevertheless, only for low intensities it was possible to suppress the H-reflex. With higher intensities of the current the H-reflex was not suppressed. On the right side of the figure an index (see Methods) quantified the amount of reduction of the EMG activation by coupling electrical and optical stimulation both at lumbar and at the thoracic level. While with the optical stimulation of the lumbar spinal cord we observed a reduction of the H-reflex, we did not observe such an effect with optical stimulation of the thoracic spinal cord. (TIF) [file pone.0100865.s002.tif]

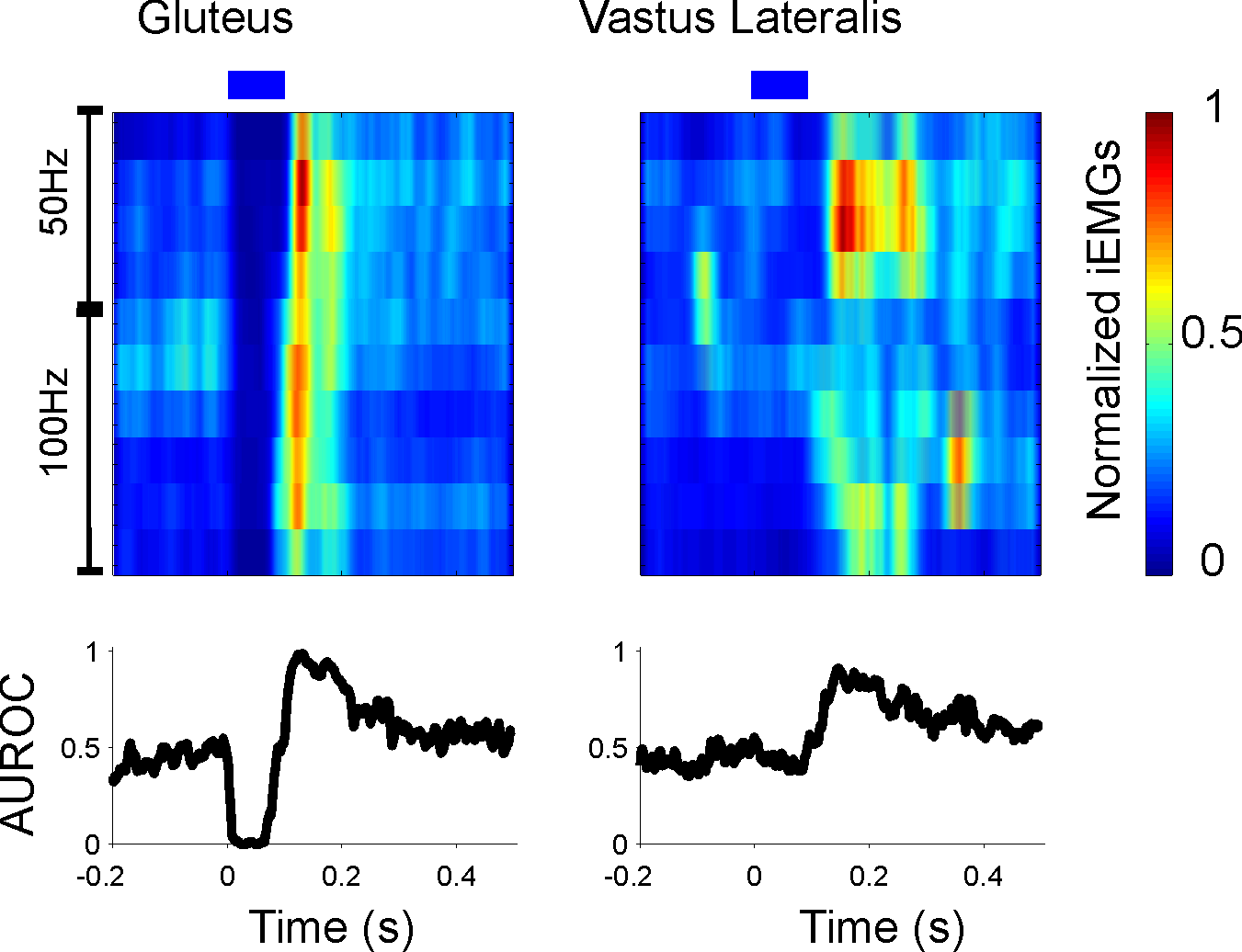

Supplement: Figure S3 — EMG and AUROC analysis in awake freely moving animals. Normalized (upper row) and AUROC EMG analysis (lower row) of simultaneously recorded muscles. Optical fiber implanted around T13-L1. Only the activity of the Gluteus was suppressed by the optical stimulation while the Vastus Lateralis was not affected. Same conventions as in Figure 5. (TIF) [file pone.0100865.s003.tif]
